# Gene copy number variation and pediatric mental health/neurodevelopment in a general population

**DOI:** 10.1093/hmg/ddad074

**Published:** 2023-05-08

**Authors:** Mehdi Zarrei, Christie L Burton, Worrawat Engchuan, Edward J Higginbotham, John Wei, Sabah Shaikh, Nicole M Roslin, Jeffrey R MacDonald, Giovanna Pellecchia, Thomas Nalpathamkalam, Sylvia Lamoureux, Roozbeh Manshaei, Jennifer Howe, Brett Trost, Bhooma Thiruvahindrapuram, Christian R Marshall, Ryan K C Yuen, Richard F Wintle, Lisa J Strug, Dimitri J Stavropoulos, Jacob A S Vorstman, Paul Arnold, Daniele Merico, Marc Woodbury-Smith, Jennifer Crosbie, Russell J Schachar, Stephen W Scherer

**Affiliations:** The Centre for Applied Genomics, The Hospital for Sick Children, Toronto, ON M5G 0A4, Canada; Program in Genetics and Genome Biology, The Hospital for Sick Children, Toronto, ON, M5G 0A4, Canada; Neurosciences and Mental Health Program, The Hospital for Sick Children, Toronto, ON M5G 0A4, Canada; The Centre for Applied Genomics, The Hospital for Sick Children, Toronto, ON M5G 0A4, Canada; Program in Genetics and Genome Biology, The Hospital for Sick Children, Toronto, ON, M5G 0A4, Canada; The Centre for Applied Genomics, The Hospital for Sick Children, Toronto, ON M5G 0A4, Canada; Program in Genetics and Genome Biology, The Hospital for Sick Children, Toronto, ON, M5G 0A4, Canada; The Centre for Applied Genomics, The Hospital for Sick Children, Toronto, ON M5G 0A4, Canada; Neurosciences and Mental Health Program, The Hospital for Sick Children, Toronto, ON M5G 0A4, Canada; The Centre for Applied Genomics, The Hospital for Sick Children, Toronto, ON M5G 0A4, Canada; Program in Genetics and Genome Biology, The Hospital for Sick Children, Toronto, ON, M5G 0A4, Canada; Neurosciences and Mental Health Program, The Hospital for Sick Children, Toronto, ON M5G 0A4, Canada; The Centre for Applied Genomics, The Hospital for Sick Children, Toronto, ON M5G 0A4, Canada; The Centre for Applied Genomics, The Hospital for Sick Children, Toronto, ON M5G 0A4, Canada; The Centre for Applied Genomics, The Hospital for Sick Children, Toronto, ON M5G 0A4, Canada; The Centre for Applied Genomics, The Hospital for Sick Children, Toronto, ON M5G 0A4, Canada; The Centre for Applied Genomics, The Hospital for Sick Children, Toronto, ON M5G 0A4, Canada; Ted Rogers Centre for Heart Research, Cardiac Genome Clinic, The Hospital for Sick Children, Toronto, ON M5G 0A4, Canada; The Centre for Applied Genomics, The Hospital for Sick Children, Toronto, ON M5G 0A4, Canada; The Centre for Applied Genomics, The Hospital for Sick Children, Toronto, ON M5G 0A4, Canada; Program in Genetics and Genome Biology, The Hospital for Sick Children, Toronto, ON, M5G 0A4, Canada; The Centre for Applied Genomics, The Hospital for Sick Children, Toronto, ON M5G 0A4, Canada; The Centre for Applied Genomics, The Hospital for Sick Children, Toronto, ON M5G 0A4, Canada; Genome Diagnostics, Department of Paediatric Laboratory Medicine, The Hospital for Sick Children, Toronto, ON M5G 0A4, Canada; Laboratory Medicine and Pathobiology, University of Toronto, Toronto, ON M5S 1A8, Canada; The Centre for Applied Genomics, The Hospital for Sick Children, Toronto, ON M5G 0A4, Canada; Program in Genetics and Genome Biology, The Hospital for Sick Children, Toronto, ON, M5G 0A4, Canada; The Centre for Applied Genomics, The Hospital for Sick Children, Toronto, ON M5G 0A4, Canada; The Centre for Applied Genomics, The Hospital for Sick Children, Toronto, ON M5G 0A4, Canada; Program in Genetics and Genome Biology, The Hospital for Sick Children, Toronto, ON, M5G 0A4, Canada; Departments of Statistical Sciences, Computer Science and Biostatistics, University of Toronto, Toronto, ON M5G 1Z5, Canada; Genome Diagnostics, Department of Paediatric Laboratory Medicine, The Hospital for Sick Children, Toronto, ON M5G 0A4, Canada; The Centre for Applied Genomics, The Hospital for Sick Children, Toronto, ON M5G 0A4, Canada; Program in Genetics and Genome Biology, The Hospital for Sick Children, Toronto, ON, M5G 0A4, Canada; Department of Psychiatry, University of Toronto, Toronto, ON M5T 1R8, Canada; Autism Research Unit, The Hospital for Sick Children, Toronto, ON M5G 0A4, Canada; Program in Genetics and Genome Biology, The Hospital for Sick Children, Toronto, ON, M5G 0A4, Canada; Mathison Centre for Mental Health Research and Education, University of Calgary, Calgary, AB T2N 1N4, Canada; Departments of Psychiatry & Medical Genetics, Hotchkiss Brain Institute, Cumming School of Medicine, University of Calgary, Calgary, AB T2N 1N4, Canada; The Centre for Applied Genomics, The Hospital for Sick Children, Toronto, ON M5G 0A4, Canada; Deep Genomics Inc., Toronto, ON M5G 1M1, Canada; The Centre for Applied Genomics, The Hospital for Sick Children, Toronto, ON M5G 0A4, Canada; Institute of Neuroscience, Newcastle University, Newcastle upon Tyne NE1 7RU, UK; Neurosciences and Mental Health Program, The Hospital for Sick Children, Toronto, ON M5G 0A4, Canada; Department of Psychiatry, University of Toronto, Toronto, ON M5T 1R8, Canada; Neurosciences and Mental Health Program, The Hospital for Sick Children, Toronto, ON M5G 0A4, Canada; Department of Psychiatry, University of Toronto, Toronto, ON M5T 1R8, Canada; Institute of Medical Science, University of Toronto, Toronto, ON M5S 1A8, Canada; The Centre for Applied Genomics, The Hospital for Sick Children, Toronto, ON M5G 0A4, Canada; Program in Genetics and Genome Biology, The Hospital for Sick Children, Toronto, ON, M5G 0A4, Canada; Department of Molecular Genetics, McLaughlin Centre, University of Toronto, Toronto, ON M5S 1A8, Canada

## Abstract

We assessed the relationship of gene copy number variation (CNV) in mental health/neurodevelopmental traits and diagnoses, physical health and cognition in a community sample of 7100 unrelated children and youth of European or East Asian ancestry (Spit for Science). Clinically significant or susceptibility CNVs were present in 3.9% of participants and were associated with elevated scores on a continuous measure of attention-deficit/hyperactivity disorder (ADHD) traits (*P* = 5.0 × 10^−3^), longer response inhibition (a cognitive deficit found in several mental health and neurodevelopmental disorders; *P* = 1.0 × 10^−2^) and increased prevalence of mental health diagnoses (*P* = 1.9 × 10^−6^, odds ratio: 3.09), specifically ADHD, autism spectrum disorder anxiety and learning problems/learning disorder (*P’s* < 0.01). There was an increased burden of rare deletions in gene-sets related to brain function or expression in brain associated with more ADHD traits. With the current mental health crisis, our data established a baseline for delineating genetic contributors in pediatric-onset conditions.

## Introduction

Many children and youth suffer from impairing and persistent mental health conditions and neurodevelopmental disorders (NDDs) such as attention-deficit/hyperactivity disorder (ADHD), obsessive–compulsive disorder (OCD) and autism spectrum disorder (ASD). There is a high degree of co-occurrence of mental health disorders and NDDs and they often present with deficits in cognition (e.g. worse response inhibition, increased response variability) (1–3). Twin, genetic and molecular studies indicate that genetic risk factors along with environmental influences create variation in neurodevelopment with disorders representing the extremes of typically widely distributed cognitive and behavioral traits that underlie mental health state.

Copy number variation (CNV) refers to deletions or duplications of segments of DNA that alter the typical diploid state of genes and/or their regulatory elements along chromosomes (4). CNV is ubiquitous in all genomes and when such genetic events (new large CNV arises ~1/100 meioses) impact important brain developmental genes it can cause or increase risk to mental health issues (5–7). ASD (8) and schizophrenia are the prototypical examples, but CNVs are also found involved in most every other brain condition analyzed (Supplementary Material, Table S1) (9). Some rare (<1% population frequency) CNVs are so strongly related to mental health disorders and NDDs that they are recognized by the American College of Medical Genetics guidelines (10) as pathogenic or likely pathogenic CNVs (clinically significant)*.*

Most studies of the relationship between rare CNVs and mental health disorders are currently based on examining clinical databases of individuals with ASD, ADHD or intellectual deficiency. There have been few general population studies of the prevalence and mental health significance of CNVs in children. However, these general population studies have tended to focus on only a narrow range of mental health conditions or NDDs, types of CNVs (typically large deletions) and have not distinguished among CNVs according to established risk (significant versus susceptibility) (11). With increasing use of genome-wide analysis in clinical care (12,13), we developed a study with the aim of advancing our understanding of CNV and the genes they impact on mental health and NDD diagnoses and traits, both behavioral and cognitive.

## Results

### Prevalence of reported mental health/NDDs diagnoses, traits and clinically significant and susceptibility CNVs

**Figure 1:**
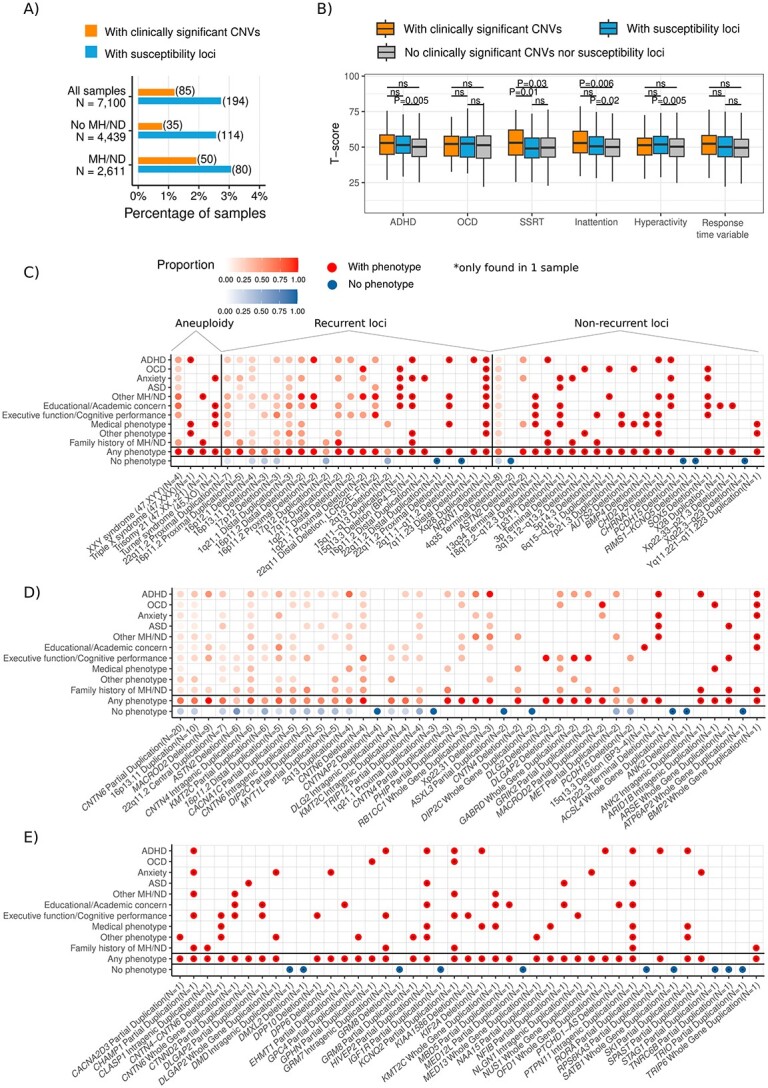
CNVs involved in mental health disorders and neurodevelopmental disorders diagnosis and traits. (**A**) proportion of samples with a mental health disorder/neurodevelopmental disorder (MH/ND) diagnosis or traits, and the proportion of each category carrying clinically significant or susceptibility CNVs, (**B**) comparison of T-scores for OCD traits, ADHD traits or SSRT and RTV traits between subjects carrying clinically significant CNVs, susceptibility CNVs, and those with neither type of CNVs, and (**C**) distribution of reported diagnosis and/or high traits of mental health/NDDs in participants carrying clinically significant CNVs, and (**D**) and (**E**) distribution of a reported diagnosis or high trait (top 10%) of a mental health disorder/NDD in participants carrying susceptibility CNVs. The number on the bars (A) indicates the number of participants carrying CNVs in each category. Abbreviations: ADHD: attention-deficit/hyperactivity disorder, OCD: obsessive–compulsive disorder, ASD: autism spectrum disorder, MH: mental health disorder, ND: neurodevelopmental disorders, SSRT: response inhibition using the Stop-Signal task. Other MH/ND: a reported diagnosis of Tics/Tourette’s, learning problem/disability, eating disorder, mood disorder, schizophrenia/bipolar/manic depressive, motor disability, intellectual disability/developmental delay and/or language disorder (s). Medical Phenotype: a reported diagnosis or treatment of medical disorders and genetic conditions. Other Phenotype: an additional phenotype not covered by the other categories that may be relevant to identified CNVs. For details refer to Supplementary Material, Table S3.

Reported diagnoses of mental health disorders/NDDs were reported in 17.5% (1232/7050) of participants and 27.6% (1948/7050) scored in the highest 10% on any of the trait measures (Fig. 1A; Table 1 and Supplementary Material, Table S2). Cumulatively, 34.2% (2412/7050) of participants reported either a mental health disorder or scored in the high-trait group (Table 1 and Supplementary Material, Table S2) and 10.9% (768/7050) had both. Learning disabilities/learning problems were the most frequently reported disorder (9.1%) followed by ADHD (6.7%). Rates of diagnoses by age and gender are shown in Supplementary Material, Table S2.

**Table 1:** Demographics and rate of mental health/neurodevelopmental disorders reported diagnoses and traits

	***N***	**(%)**
**Total sample**	**7100**	
**Gender**		
Male	3558	(50.1%)
Female	3542	(49.9%)
**Age (mean, CI) *years***	10.7	[4.9–16.5]
4.0–11.9	4627	(65.2%)
12.0–19.0	2473	(34.8%)
**Ancestry**		
European	5686	(80.1%)
East Asian	1414	(19.9%)
**Reporting source**		
Parent report	5956	(83.9%)
Self report	1144	(16.1%)
**CNV type**		
Significant	85	(1.2%)
Susceptibility	194	(2.7%)
No significant or susceptibility CNV	6821	(96.1%)
**Self-reported diagnosis**	***n* = 7050** ^b^	
ADHD	474	(6.7%)
Anxiety	320	(4.5%)
ASD	145	(2.1%)
Eating disorder	2	(0.03%)
Intellectual disability/developmental delay	13	(0.2%)
Language disorder	5	(0.1%)
Learning disorder/learning problem	644	(9.1%)
Mood disorder	96	(1.4%)
Motor delay/motor disability	4	(0.1%)
OCD	80	(1.1%)
Tics/Tourette’s	84	(1.2%)
Schizophrenia/bipolar/manic depressive	6	(0.1%)
VCFS	1	(0.0%)
Any diagnosis	1232	(17.5%)
Any high trait^a^	1948	(27.6%)
Any diagnosis or high trait	2412	(34.2%)

Abbreviations: *N*: number of samples in each designated category; ADHD: attention-deficit/hyperactivity disorder; SWAN: The Strengths and Weaknesses of Attention-Deficit/Hyperactivity Disorder Symptoms and Normal Behavior Scale; OCD: obsessive–compulsive disorder; TOCS: Toronto Obsessive–Compulsive Scale; ASD: autism spectrum disorder, AQ: Autism Spectrum Quotient; RCADS: Revised Children’s Anxiety and Depression Scale; CBCL: Child Behavior Checklist; VCFS: velocardiofacial syndrome (see Supplementary Text for details).

^a^High traits: Top 10% traits of ADHD (SWAN; total score, inattentive or hyperactive/impulsive), obsessions/compulsions (TOCS), anxiety (CBCL; Spit for Science 1), mood and anxiety (RCADS; Spit for Science 2), autism (AQ; Spit for Science 2) *t*-scores. Total cases with available trait scores are as follows: ADHD (7023), OCD (7010), Anxiety (5607), ASD (1787).

^b^Overall *n* used is restricted to those that have diagnosis and trait data (Total *n* = 7050, 5660 of European Ancestry, and 1390 of East Asian Ancestry). Participants could have multiple diagnoses and belong to multiple high-trait groups.

Using criteria specified in Supplementary Materials, we identified 287 (3.9%) clinically significant or susceptibility CNVs in 279 of 7100 individuals across both European and East Asian ancestry cohorts (Table 2; Supplementary Material, Table S3; Fig. 1A; Supplementary Material, Fig. S1). Of these, 1.2% (85/7100) of participants harbored clinically significant CNVs and 2.7% (194/7100) carried susceptibility CNVs. Participants of European (223/5686 = 3.9%) and East Asian (56/1414 = 4.0%) ancestries had similar rates of clinically significant and/or susceptibility CNVs (*ns*). There were also no statistically significant differences by age, sex or respondent (Age: clinically significant/susceptibility = 10.96 years, neither CNV = 10.68 years, *ns;* Sex: clinically significant/susceptibility = 45.5% female, neither CNV = 50% female, *ns*; Respondent: clinically significant susceptibility = 83.5% parent-report, neither CNV = 83.9% parent-report, *ns*).

**Table 2:** CNV characteristics

Category	Spit for Science			ALSPAC (Replication Cohort)		
	EUR	EAS	All	EUR	non-EUR	All
Clinically significant CNVs	75 (1.3%)	10 (0.7%)	85 (1.2%)	70 (1.8%)	77 (2.3%)	147 (2.0%)
A: Aneuploidy	7 (0.1%)	0 (0%)	7 (0.1%)	0 (0%)	1 (0.03%)	1 (0.01%)
B: Recurrent CNVs	43 (0.8%)	5 (0.3%)	48 (0.7%)	40 (1.0%)	46 (1.4%)	86 (1.2%)
C: Non-recurrent CNVs	25 (0.4%)	6 (0.4%)	31 (0.4%)	30 (0.8)	30 (0.9%)	60 (0.8%)
Susceptibility CNVs	148 (2.6%)	46 (3.3%)	194 (2.7%)	54 (1.4%)	51 (1.5%)	105 (1.5%)
Clinically significant or susceptibility CNVs	223 (3.9%)	56 (3.9%)	279 (3.9%)	123 (3.2%)	127 (3.8%)	250 (3.5%)

Distribution of different clinically significant and susceptibility CNVs identified in 7100 samples in Spit for Science (*n* = 5686 for European and 1414 for East Asian ancestry) and a replication cohort of 7219 samples from the Avon Longitudinal Study of Parents and Children study (*n* = 3890 for European and 3329 in non-European ancestry).

Abbreviations: OSC: Spit for Science; EUR: European; EAS: East Asian; ALSPAC: Avon Longitudinal Study of Parents and Children; All: all ethnicities.

Numbers outside parentheses indicate the raw count of samples with CNVs in the indicated CNV category. Digits inside parentheses signify frequency. Clinically significant CNVs were those classified as pathogenic or likely pathogenic, which were stratified into three classes: aneuploidy, recurrent CNVs and non-recurrent CNVs. Susceptibility CNVs referred to variants with evidence linking them to neurodevelopmental disorders, but with insufficient evidence to establish their pathogenicity. We analyzed CNVs greater than 50 kb in size for the ALSPAC cohort, whereas a minimum size cut-off of 10 kb was used OSC CNVs (see the main text and the Supplementary Information for details). CNV calls for sex chromosomes for this cohort were not available for ALSPAC**. Statistics for all samples in the OSC co**hort with CNVs over 50 kb are: clinically significant CNVs: 84 (1.2%); aneuploidy: 7 (0.1%); recurrent CNVs: 48 (0.7%); non-recurrent CNVs: 29 (0.4%); susceptibility CNVs: 184 (2.6%); clinically significant or susceptibility CNVs: 268 (3.8%**). We confirmed the validity of these variants using qPCR or by visualizing the B-allele frequency.**

### Similar rates of clinically significant and/or susceptibility CNVs findings in an independent pediatric cohort

As shown in Table 2, we found clinically significant and/or susceptibility CNVs in 3.5% (250/7219) of Avon Longitudinal Study of Parents and Children (ALSPAC) participants ranging from 3.2% in European ancestry to 3.8% in non-European ancestry participants (Supplementary Material, Table S4). The distribution of significant CNVs and susceptibility CNVs among European and non-European ancestry participants is similar to that of the Spit for Science samples (Supplementary Material, Table S4). A previous study found that 1.25% of participants carried a clinically significant and/or susceptibility CNV in ALSPAC (14), but analyzed only a subset of those type of variants.

### Clinically significant and/or susceptibility CNVs increase likelihood of mental health disorders/NDD reported diagnoses or high traits

Of the 279 participants with clinically significant or susceptibility CNVs in the Spit for Science cohort, 28% (78/279) reported a diagnosis of a mental health disorder/NDD (MH/ND), 38.7% (108/279) met criteria for membership in one of the high-trait groups and 46.6% (130/279) had either a diagnosis or high traits (Table 3; Supplementary Material, Table S2). We found that 59 and 41% of participants with clinically significant and susceptibility CNVs respectively had reported a diagnosis or high traits (Table 3) compared with 34% of participants without either type of CNV.

**Table 3:** Enrichment of mental health disorder/neurodevelopmental disorder diagnoses and traits among participants with clinically significant, susceptibility or either type of CNV

**Category**	**Count**	**Reported Dx (All MP/ND)** ^**a**^			**High Traits** ^**b**^			**Reported Dx *and/or* High Traits**		
		**YES (%)**	**NO (%)**	**OR**	**YES (%)**	**NO (%)**	**OR**	**YES (%)**	**NO (%)**	**OR**
**Clinically significant CNVs**	85	33 (39%)	52 (61%)	3.09^*^^*^^*^^*^	39 (46%)	46 (54%)	2.27^*^^*^^*^	50 (59%)	35 (41%)	2.81^*^^*^^*^^*^
**No significant or susceptibility CNV**	6771	1154 (17%)	5617 (83%)		1840 (27%)	4931 (73%)		2282 (34%)	4489 (66%)	
***Total***	*6856*	*1187 (17%)*	*5669 (83%)*		*1879 (27%)*	*4977 (73%)*		*2332 (34%)*	*4524 (66%)*	
**Susceptibility CNVs**	194	45 (23%)	149 (77%)	1.47^*^	69 (36%)	125 (64%)	1.48^*^^*^	80 (41%)	114 (59%)	1.38^*^
**No significant or susceptibility CNV**	6771	1154 (17%)	5617 (83%)		1840 (27%)	4931 (73%)		2282 (34%)	4489 (66%)	
***Total***	*6965*	*1199 (17%)*	*5766 (83%)*		*1909 (27%)*	*5056 (73%)*		*2362 (34%)*	*4603 (66%)*	
**Clinically significant or Susceptibility CNVs**	279	78 (28%)	201 (72%)	1.89^*^^*^^*^^*^	108 (39%)	171 (61%)	1.69^*^^*^^*^^*^	130 (47%)	149 (53%)	1.72^*^^*^^*^^*^
**No significant or susceptibility CNV**	6771	1154 (17%)	5617 (83%)		1840 (27%)	4931 (73%)		2282 (34%)	4489 (66%)	
***Total***	*7050*	*1232 (17%)*	*5818 (83%)*		*1948 (28%)*	*5102 (72%)*		*2412 (34%)*	*4638 (66%)*	

^*^ *P* < 0.05; ^*^^*^P < 0.01; ^*^^*^^*^P < 0.001; ^*^^*^^*^^*^P < 0.00001. OR (odds ratios) using Chi-square test for independence, in relation to not having significant or susceptibility CNV. (%): Row percentages; Totals are in *italics*; Odds ratios are in **bold**.

^a^Reported diagnosis (DX): ADHD, OCD, ASD, anxiety, tics/Tourette’s, learning problem (OSC1)/disability (OSC2), eating disorder, mood disorder, Schizophrenia, bipolar, motor disability (OSC1, OSC2Summer), velocardiofacial syndrome, intellectual disability (OSC1 & OSC2)/developmental delay (OSC1), language disorder (OSC1, OSC2 Summer)

^b^High traits: top 10% gender adjusted *t*-scores for SWAN (ADHD), TOCS (OCD), CBCL (Anxiety, OSC1), RCADS (Anxiety, OSC2) and/or AQ (ASD, only asked in OSC2). People with reported diagnoses were not excluded from the high-trait group if they met the definition. OSC1 = first phase of sample collection; OSC2 = second phase of sample collection. See Supplementary Material for details.

Participants carrying either a significant or susceptibility CNV compared with those with neither type of CNV were 3.1–1.7 times more likely to report a mental health disorder/NDD or have high-trait scores (Table 3). Mental health disorders were more likely among individuals carrying clinically significant CNVs [*p* = 1.9 × 10^−6^, odds ratio: 3.09 (CI:1.92–4.89)], susceptibility CNVs [*P* = 0.03, odds ratio: 1.47 (CI:1.02–2.08)] or either type of CNVs [*P* = 8.46 × 10^−6^, odds ratio: 1.89 (CI:1.42–2.48)] compared with those who did not have any clinically significant or susceptibility CNVs (Table 3). High traits were also significantly more likely across both CNV types (Table 3), highlighting the utility of using trait-based mental health phenotyping to detect genetics associations. Furthermore, participants with a reported diagnosis and/or high traits of, mental health disorders/NDDs were also more likely than those without these phenotypes to have a clinically significant or susceptibility CNV (5.9% vs. 3.5%; *P* < 0.0001; see Table 3).

As shown in Figure 1B, participants with susceptibility CNVs had significantly higher traits of ADHD (*P* = 0.005), inattention (*P* = 0.02) and hyperactivity (*P* = 0.005) than those with neither clinically significant or susceptibility CNVs. Participants with clinically significant CNVs had greater inattention scores (*P* = 0.006) and worse response inhibition (SSRT, *P* = 0.03) than those neither clinically significant or susceptibility CNVs (*P* = 0.006). Diagnosis of ADHD, Anxiety, ASD and learning problems/learning disorder were more prevalent in participants with clinically significant or susceptibility CNVs compared to those with neither (*P* < 0.01; Table 4)

**Table 4:** Mental health disorder/neurodevelopmental disorder diagnoses in participants with and without clinically significant or susceptibility copy number variants

	**Clinically Significant or Susceptibility CNV *n* = 279**		**Neither Type of CNV *n* = 6771**		***P*-value**
**Disorders (Frequency, %)**					
ADHD	31	11.1%	443	6.5%	**0.003**
Anxiety	22	7.9%	298	4.4%	**0.007**
ASD	15	5.4%	130	1.9%	**< 0.0001**
Eating disorder	0	0.0%	2	0.0%	-
Intellectual disability/developmental delay	3	1.1%	10	0.1%	-
Language disorder	0	0.0%	5	0.1%	-
Learning disorder/learning problem	47	16.8%	597	8.8%	**< 0.0001**
Mood disorder	5	1.8%	91	1.3%	ns
Motor delay/motor disability	1	0.4%	3	0.0%	-
OCD	3	1.1%	77	1.1%	ns
Schizophrenia/bipolar/manic depressive	0	0.0%	6	0.1%	-
Tics/Tourette’s	6	2.2%	78	1.2%	ns
VCFS	1	0.4%	0	0.0%	-

Disorders with a total *n* < 20 were not included in logistic regression analyses (− in *P*-value column). ADHD = attention-deficit/hyperactivity disorder, ASD = autism spectrum disorder, OCD = obsessive–compulsive disorder, VCFS = velocardiofacial syndrome; Significant p-values are in **bold**.

### Clinically significant and susceptibility CNVs confer risk across mental health phenotypes

We presented all clinically significant CNVs and associated phenotypes in Figure 1C and all susceptibility CNVs in Figure 1D and E (see Supplementary Material, Table S3 for details). Variable expressivity and pleiotropy of a CNV (15) were implied by the observation of different phenotypes associated with the respective CNV. We found aneuploidies in 0.1% (*n* = 7) of European ancestry participants, but none in East Asian ancestry group: one female trisomy 21, one triple X syndrome (47, XXX), four XYY syndromes, and one female 45, XO participant. Individuals with XYY syndrome showed a spectrum of different disorders and traits, notably ADHD, ASD, OCD, anxiety and learning problems, whereas the triple X syndrome participants did not report any MH/ND phenotype though they reported Celiac Disease (Fig. 1C).

Recurrent CNVs (i.e. the same rearrangements that arise independently in the population) were found in 0.7% (48/7100) of participants (Table 2 and Supplementary Material, Table S3; Fig. 1C). Examples of these CNVs that were associated with a spectrum of different phenotypes are 15q11-q13 duplication, associated with ASD, OCD, learning problem and anxiety, 22q11.2 deletion, associated with ASD, ADHD and learning problem and 16p12.1 distal duplication syndrome with anxiety disorder (Fig. 1C). CNVs impacting *NRXN1* (with ADHD, OCD, anxiety traits, ASD, executive function and learning problems), *ASTN2* in male (with ADHD, anxiety and seizure) and *CHD2* (ADHD, learning problems, epilepsy and hearing problems) were examples of non-recurrent clinically significant CNVs associated with phenotypes (Fig. 1C; Table 2; Supplementary Material, Table S3). Terminal 4q35 deletion, Xq22.3-q23 deletion and deletions impacting *PCDH15*, and *KCNQ5* from this class, did not show any phenotype in our cohort.

Examples of susceptibility CNVs were deletions and duplications impacting *MACROD2* (16) (with ADHD, anxiety, learning problem, response inhibition and reaction time variability (RTV) phenotype and family history of mental health condition), *DLGAP2* (with ADHD, anxiety, reaction time phenotype and family history of mental health condition), *DLG2* (reaction time phenotype), *DPP6* (with anxiety traits), *GRIK2* (with ADHD, reaction time and response inhibition phenotypes and family history of mental health condition) and *CHAMP1* (with ADHD, anxiety, learning problems and family history of mental health condition) (Fig 1C; Table 2; Supplementary Material, Table S3). Of the latter class, the following CNVs were examples that did not manifest any phenotype: deletions or duplications on *CNTNAP2*, *ASXL3*, *ANK2*, *MBD5*, *DMXL2* and *TRIO*. However, the participant with the CNV on *MBD5* duplication is reported as being gifted and attends a special classroom.

We found 35 unrelated participants carrying duplications impacting either *CNTN6*, *CNTN4* or both genes. Sixteen of these participants reported a mental health condition or high traits (Supplementary Material, Table S3). We performed whole-genome sequencing of 19 samples to search for variants associated with NDDs which might have been missed by array (see Supplementary Material), but none were found.

We identified eight participants carrying multiple clinically significant or susceptibility CNVs (Supplementary Material, Table S3). One male participant of East Asian ancestry had a 28.9 kb duplication overlapping *DLG2* and a 90.3 kb duplication overlapping *TRIP12*, had slowed reaction time but no reported mental health/NDD phenotype. A female participant of European descent had two recurrent genomic disorder CNVs: a 16p11.2 proximal duplication (604 kb) and a 1q21.1 distal duplication (1.05 Mb). She had a family history of ADHD. We also analyzed for the presence of clinically significant and susceptibility SNVs but nothing compelling was found (Supplementary Material, Table S3).

### CNV burden associated with mental health disorder/NDD traits

#### Overall burden

The number of genes impacted by rare deletions was positively correlated with inattention traits in the European ancestry participants [*β* = 0.15, 95% CI = (0.01, 0.3), *P* = 0.04]. We did not find any association between the rare or less rare CNVs and other traits in Europeans or East Asians. Although previous studies found an enrichment of deletions in ADHD (17–20), we found a non-significant trend for this association with ADHD traits in our sample [*β* = 0.12, 95% CI = (−0.03, 0.26), *P* = 0.12]. Although a relationship between ADHD and low IQ and between low IQ and large CNVs has been previously described (21), low IQ is not likely driving the increase in large CNVs for ADHD in our study. Although we did not measure IQ, there were only two cases with reported intellectual or developmental delay and thus were not likely strong contributors to the findings. Consistent with previous findings with OCD patients, we did not observe association with duplications or deletions (22) (Supplementary Material, Table S5). There were no sex differences in the total size of deletions/duplications and the total number of genes impacted by deletions/duplications (Supplementary Material, Table S5).

#### Gene-set (pathway) burden

**Figure 2:**
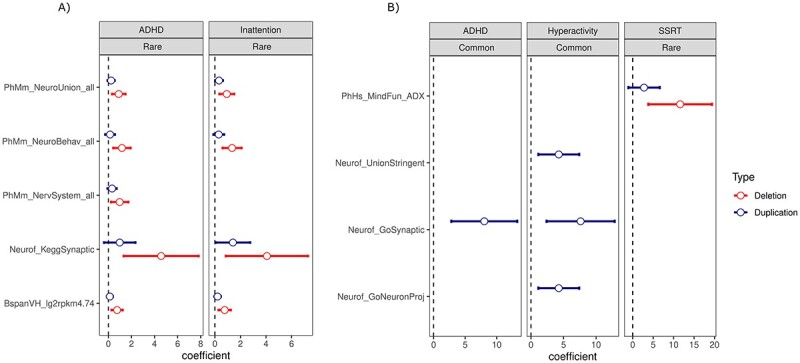
Gene-set burden analysis. Associated gene-sets identified by gene-set burden analysis of rare (< 0.05 frequency) and common (1.0–5.0 frequency) CNVs separately for deletions and duplications in (**A**) European and (**B**) East Asian ancestry participants. The dot represents the beta coefficient from the regression model, and the arm represents 95% confidence intervals of the coefficient. See Supplementary Material, Table S5 for details on gene-sets. All phenotypes presented in gray boxes are trait scores.

In the European ancestry group, we found an increased burden of genes impacted by rare deletions that are highly expressed in brain (*P* = 0.02, BH-FDR = 0.15), or are in synaptic pathway (*P* = 0.03, BH-FDR = 0.15), mice neuronal behavior (*P* = 0.03, BH-FDR = 0.15) and mice nervous system (*P* = 0.05, BH-FDR = 0.20) in ADHD traits (Fig. 2; Supplementary Material, Fig. S2, Fig. S3; Supplementary Material, Table S5). A similar association was observed with deletions in inattention traits. In the East Asian ancestry group, we found a significant association between the burden of rare duplications impacting genes related to higher mental function and SSRT (*P* = 5 × 10^−3^, BH-FDR = 0.11). ADHD and hyperactivity traits were associated with synaptic or neurofunctional genes in the analysis of common deletions (*P* < 0.05, BH-FDR < 0.2).

Burden analysis of genes within chromosome X was also performed for individuals of European ancestry. We found a significant association between rare deletion impacting genes that are highly expressed in brain for ADHD traits (*P* = 0.02, BH-FDR = 0.11), inattention traits (*P* = 0.04, BH-FDR = 0.18) and hyperactivity traits (*P* = 0.04, BH-FDR = 0.18).

#### Locus burden & specific genes driving burden

No loci passed the genome-wide significant threshold (Family-wiser error rate—adjusted *P* < 0.05, Supplementary Material, Table S5). In the European ancestry subset, we found that rare deletions impacting *ADCYAP1*, *BBOX1*, *CSMD1*, *ZNF74*, *DGCR6*, *BCR* and *GRM6* were associated with ADHD traits. Similarly, inattention traits were associated with *BBOX1*, *CSMD1* and *GRM6* for deletions and 22q11.21 for both deletions and duplications. For the analysis of X-linked genes, we also found rare deletions impacting Xp22.31 region (*PNPLA4* and *STS*) were associated with ADHD traits, inattention traits and hyperactivity-impulsivity traits. For East Asian subset, we found that 15q11.2 duplication (*CYFIP1*) was significantly associated with ADHD traits and hyperactivity-impulsivity traits.

## Discussion

Our findings demonstrate the value of a community-based sample to help to clarify the role of genetic variation in mental health and neurodevelopment. First, we showed that CNVs that are classified as pathogenic based on clinical studies often co-occur with mental health and neurodevelopmental (ND) phenotypes in the general population, but not always. There are several reasons why there might be no phenotype in individuals with a putative pathological CNV. For example, participants might be too young to manifest adult- or adolescent-onset disorders (23,24), failure to report for personal reasons in the context of a community study, bias in which people access clinical care (25) or they have CNVs with incomplete penetrance (4,12,26–28). Although we were not able to clinically verify diagnoses, the prevalence of reported disorders (e.g. ADHD 6.7%) in our sample was similar with existing population figures (29,30). The overall prevalence of diagnoses of mental health disorders in our Spit for Science cohort i.e. 17.5% in Table 1, was also in accordance with that in the Sweden study (20.9%) (31) but less than that (36.7%) in the North Carolina study (32). The Sweden study estimate did not include anxiety and depression, which we did account for.

Second, we found clinically significant CNVs with various manifestation of mental health disorders/NDDs, mainly ADHD, OCD, ASD reported diagnoses and related behavior and cognitive traits (response inhibition). These findings add to mounting evidence that traits share genetic risk with their respective disorders (33,34). Our approach also identified novel variants for each disorder (Supplementary Material, Table S5), showing the added value of the trait-based approach. Another benefit of trait-based measurement is the ability to examine cognitive deficits present across many psychiatric disorders. Interestingly, response inhibition, which is a common cognitive deficit in ADHD and shares genetic risk with the disorder (35,36), showed overlap in burden findings and specific loci. Many of the CNVs associated with ADHD, OCD and response inhibition traits have been previously implicated in other ND studies (11,13). Our new findings support evidence from previous studies of common and rare variants of pleiotropy among these disorders (37–41).

The current study observed a distribution of clinically significant and/or susceptibility CNVs much like those reported in previous child and adult population studies i.e. ALSPAC (14), The Child and Adolescent Twin Study in Sweden (31) and The Norwegian Mother, Father and Child study (42). Other studies focused on adults i.e. UK Biobank (26,43), Estonian Genome Centre at the University of Tartu (28), Minnesota Centre for Twin and Family Research (28) and Geisinger MyCode community health initiative (44) or children and adults (BioMe Biobank) (45) have been previously published. However, all these studies focused on recurrent and/or non-recurrent contiguous genomic syndromes only. Several studies examined CNVs in two well-known pathogenic, genes linked to i.e. *NRXN1* and *SHANK3*, (summary of afore-mentioned studies in Supplementary Material, Table S6) (14,26). Our study was unique in that it evaluated the clinical impact of every CNV including those impacting genes associated with various mental health disorders and NDDs, which leads us to find all possible clinically significant and susceptibility CNVs. We explored variable expressivity, pleiotropy (46) and the degree of penetrance of each variant because we measured broad and comprehensive phenotypes for participants compared with aforementioned studies. Our phenotype information encompassed various child mental health disorders as well as medical conditions including diabetes, scoliosis and ulcerative colitis.

Results from community-based recruitment should be considered in the context of potential limitations. For example, recruitment at a science museum may limit participation of individuals of lower socioeconomic status (SES), those too severely affected or unable to tolerate that type of environment [e.g. Tint *et al.* (47)]. The Ontario Science Centre has programs in place to help facilitate access to lower income communities and individuals with sensory over-stimulation to help ensure equitable access. Furthermore, our recruitment relied on reported diagnosis information given the infeasibility of clinician-based diagnosis in a study of this scale in the community. However, our results suggest that the Spit for Science is broadly representative of the community at large. We have reported that although there is a slight bias toward participants with higher SES, it is relatively small (35). Additionally, the rates of reported mental health conditions are comparable to rates from large epidemiological studies [ Table 1 and Burton *et al.* (48)]. We also observed expected age and gender differences in the prevalence of reported diagnoses including a male bias in ADHD and ASD (49) and increased prevalence with age of mood and anxiety disorders (50). Furthermore, the use of quantitative trait measures also allowed us to capture individuals who may not have received a diagnosis as a result of ascertainment bias in clinic-based research which can also skew toward higher SES and more severely affected individuals (25,51). A focus on community rather than clinic-based recruitment also allowed us to potentially identify individuals with clinically significant CNVs in individuals without a phenotype (9,52).

As we have contemplated, somewhat uniquely amongt the medical fields, diagnosis in psychiatry and mental health depends largely on descriptive signs and symptoms, rather than the use of biomarkers or objective measures (53). Here we show specific CNV impacting important ND genes is found in ~4% of individuals in our community-based sample and almost 50% of those with a clinically significant or susceptibility CNV had a mental health disorder/NDD phenotype (1.7 times more likely to have that phenotype than those without either type of CNV). From our other research, we anticipate that the clinical findings will nearly double when genome sequencing is used, as all classes of genetic variants (sequence-level mutations, smaller CNVs, structural variations and mitochondrial) are found (54,55). Moreover, including polygenic risk score analysis of common genetic variants (9,56,57) or gene conservation weighting (11,58) may help to further delineate phenotypic expression of traits in penetrant CNV carriers. With the number of people who receive a clinical diagnosis of a mental health and NDDs on the rise and new CNVs/genes constantly being implicated in these conditions, irrespective of possible confounding variable expressivity and penetrance, we believe there is value to actively integrate the genomic etiological information (Fig. 1) into the clinical diagnosis paradigm.

## Materials and Methods

### Participants and measures

We examined the prevalence of rare CNVs and their associated phenotypes in a large community-based Spit for Science sample (35) of European and East Asian ancestry children and youth (*N* = 7100; 4–18 years of age; mean = 10.7 ± 2.9). Participants and their parents were visitors to the Ontario Science Centre. The Research Ethics Board of The Hospital for Sick Children gave ethical approval for this work (#1000062807 and #1000011040). Informed consent, and verbal assent where applicable, was obtained from all participants and/or their legal representatives to take part in this study and to have this research work published. The sample had a male to female sex ratio of 1.01 (male: 3558; female: 3542; Table 1 and Supplementary Material, Table S2). We focused on the two largest ancestry populations: European (*n* = 5686; M:F = 1.04) and East Asian (*n* = 1414; M:F = 0.88) for a total of 7100 participants (7050 with diagnosis and at least one trait total score available). We obtained parent or self-reported information about whether the participants had been diagnosed or treated for mental disorder, NDD/or and physical health condition. We also used reliable and valid rating scales to measure traits associated with ADHD (inattention and hyperactivity-impulsivity), OCD, anxiety and ASD (see Supplementary Materials). Participants performed a cognitive task, the stop-signal task (SST), to measure the response inhibition (stop-signal reaction time—SSRT) and reaction time variability (RTV). Longer SSRT indicates worse inhibitory control, and greater RTV indicates variability in performance. SSRT and RTV are putative cognitive biomarkers for mental health/ND disorders (1,35,36). We categorized each participant as having a ‘high trait score’ if their scores within the most extreme 10% of the sample as well as examining continuous trait measures.

### Genomic data and CNV calling/classification

DNA was extracted from saliva samples, and genotyping was performed on Illumina Infinium HumanCoreExome beadchips (5220/7100 = 73.5%) or Illumina Infinium Global Screening Array (1888/7100 = 26.6%; Supplementary Material, Table S2) (59). A high-quality set of CNVs was identified as those called by at least two of three algorithms (60). Variant size ranged from 10 kb to sex chromosome aneuploidies (*n* = 7) and a trisomy of chromosome 21 (Table 1). The relevant genotype data and CNVs are available at European Genome-Phenome Archive (accession number EGAS00001006659) and the dbVar Database (accession number nstd224), respectively. Experimental details are available as Supplementary Materials.

Clinically significant CNVs were those classified as pathogenic or likely pathogenic (see Supplemental Materials), which were stratified into three classes: aneuploidy, recurrent CNVs and non-recurrent CNVs. Susceptibility CNVs referred to variants with evidence linking them to NDDs, but with insufficient evidence to establish their pathogenicity (10,56,60). We also identified clinically significant SNVs (see Supplementary Material for details).

### Analyses

#### Sample characteristics

We examined the overall prevalence of mental health disorder and NDDs diagnoses and traits in our sample. We tested for age (< 12 years or age vs. ≥ 12 years of age), gender (male vs. female) and age × gender interactions for each disorder using logistic regression (excluding disorders with a total *n* < 20).

##### Clinically significant and susceptibility CNVs

We examined the prevalence of clinically significant and susceptibility CNVs and tested if age (in years), sex (male, female) and respondent (parent/self) differed by CNV type using one-way ANOVA and Chi-squared tests for continuous and categorical variables, respectively. We examined the rate of clinically significant and susceptibility CNV findings in an independent pediatric cohort of 7219 participants (7–17 years of age) of the Avon Longitudinal Study of Parents and Children (ALSPAC; see Supplementary Text; 3990 European and 3329 non-European ancestry) (61–63). ALSPAC is a longitudinal birth cohort, where the participants had been followed to adulthood.

We used Chi-square tests to examine if reported any mental health/NDD diagnosis and/or high traits were enriched among participants with clinically significant, susceptibility or either type of CNV. We used binary logistic regression to examine the frequency of each disorder by CNV group (clinically significant or susceptibility vs. neither). We did not include disorders with a total *n* < 20 in these analyses. We conducted linear regression to test if participants carrying clinically significant or susceptibility CNVs differed in ADHD traits total score, inattention, hyperactivity, OCD, response inhibition (SSRT) or RTV scores from those not carrying neither clinically significant or susceptibility CNVs. We also examined the type of mental health/NDD phenotypes present in different types of clinically significant and susceptibility CNVs. We sub-grouped clinically significant CNVs into aneuploidies, recurrent genomic syndromes and non-recurrent CNVs (8,60).

#### Genetic burden analysis of CNVs

##### Overall burden

To investigate the relationship of rare CNV variants with mental health traits more generally in Spit for Science, we analyzed rare (<0.5% frequency) and less-rare (1–5% frequency) variants burden using linear regression for number of genes impacted by deletions and duplications, with each trait as the outcome variable and correcting for population stratification, genotyping batch and genotyping platforms (see Supplementary Methods). Common CNVs (frequency > 5%) were not included in the analysis because of the limited number of variants. For SSRT and RTV, we also accounted for concurrent stimulant medication treatment. European and East Asian ancestry participants were analyzed separately because of unbalanced participants ratio (European/Asian = 4.0). Trait measurements in Spit for Science and ALSPAC were not comparable. Thus, no further burden analysis was performed on the latter cohort.

NDDs have been known to have a sex bias skewed toward males (49). Female protective effect concept was proposed to explain such a phenomenon; thus, this means females would require more risk variants than males to reach the disease threshold. An interesting question would be to test to see if such a bias exists in the pediatric control samples. Therefore, on top of phenotype-CNV association analysis, we also compared the burden of CNVs between sexes. A likelihood ratio test was performed to test whether total length of CNVs (deletions or duplications) and total gene count impacted by CNVs are different between males and females. Population structure (PC1–3), genotyping plate and study phase were used as covariates.

##### Gene-set (pathway) burden analysis of CNVs

We performed burden analysis of rare and less-rare CNVs on 34 gene-sets, representing human neural function and phenotype (*n* = 17), human brain gene/protein expression (*n* = 7), orthologs of mouse genes implicated in nervous system and behavioral phenotypes (*n* = 3) or other organ system phenotypes (*n* = 7); the latter can be treated as putative negative controls (Supplementary Material, Table S5). Gene-set burden was adjudicated using only gene counts per participant and was corrected for global burden (total gene count impacted by deletions and duplications) to avoid nonspecific results, following established practices (64).

##### Locus burden tests and genes driving signals in gene-set burden analysis

To identify specific rare CNVs associated with mental health disorders or traits, we performed a locus association test on the genes within the top associated gene-sets for each trait (*P* < 0.05, PH-FDR < 0.2). We present genes with deletions or duplications found in at least three individuals and *P* < 0.05 for each trait in Table S5.

## Supplementary Material

HMG-2023-CE-00137_Scherer_Supplementary_Materials_ddad074

Supplementary_Table_S1_ddad074

Supplementary_Table_S2_updated_ddad074

Supplementary_Table_S3_ddad074

Supplementary_Table_S4_ddad074

Supplementary_Table_S5_editedOSC_updated_ddad074

Supplementary_Table_S6_ddad074

## Data Availability

The relevant genotype data and CNVs are available at European Genome-Phenome Archive (accession number EGAS00001006659) and the dbVar Database (accession number nstd224), respectively. Experimental details and all other data are available in the Supplementary Materials. All scripts used in the analysis of this paper are available here: https://zenodo.org/badge/latestdoi/631017083.
